# Applications of artificial intelligence-powered prenatal diagnosis for congenital heart disease

**DOI:** 10.3389/fcvm.2024.1345761

**Published:** 2024-04-24

**Authors:** Xiangyu Liu, Yingying Zhang, Haogang Zhu, Bosen Jia, Jingyi Wang, Yihua He, Hongjia Zhang

**Affiliations:** ^1^ School of Biological Science and Medical Engineering, Beihang University, Beijing, China; ^2^ Key Laboratory of Data Science and Intelligent Computing, International Innovation Institute, Beihang University, Hangzhou, China; ^3^ State Key Laboratory of Software Development Environment, Beihang University, Beijing, China; ^4^ School of Computer Science and Engineering, Beihang University, Beijing, China; ^5^ School of Biological Sciences, Victoria University of Wellington, Wellington, New Zealand; ^6^ Echocardiography Medical Center Beijing Anzhen Hospital, Capital Medical University, Beijing, China; ^7^ Maternal-Fetal Medicine Center in Fetal Heart Disease, Beijing Anzhen Hospital, Beijing, China; ^8^ Beijing Lab for Cardiovascular Precision Medicine, Beijing, China

**Keywords:** congenital heart disease, artificial intelligence, prenatal diagnosis, fetal echocardiography, deep learning

## Abstract

Artificial intelligence (AI) has made significant progress in the medical field in the last decade. The AI-powered analysis methods of medical images and clinical records can now match the abilities of clinical physicians. Due to the challenges posed by the unique group of fetuses and the dynamic organ of the heart, research into the application of AI in the prenatal diagnosis of congenital heart disease (CHD) is particularly active. In this review, we discuss the clinical questions and research methods involved in using AI to address prenatal diagnosis of CHD, including imaging, genetic diagnosis, and risk prediction. Representative examples are provided for each method discussed. Finally, we discuss the current limitations of AI in prenatal diagnosis of CHD, namely Volatility, Insufficiency and Independence (VII), and propose possible solutions.

## 1 Introduction

Congenital heart disease (CHD) is a common and serious congenital anomaly worldwide, representing the primary contributor to infant mortality associated with birth defects (1–3). Globally, the prevalence of CHD at birth currently is 9.4‰ (2), translating to approximately one new case every three minutes. Therefore, achieving a timely and precise prenatal diagnosis of CHD is imperative, laying the foundation for informed perinatal decision-making and significantly impacting rates of morbidity and mortality (4, 5). Furthermore, prenatal genetic diagnosis of CHD holds profound implications for clinical management, prognosis, genetic counseling, and preventive measures for subsequent pregnancies.

Despite the considerable advances in prenatal diagnostics and management strategies for CHD, it remains the leading cause of neonatal mortality (6, 7). The existing gap in prenatal diagnosis can be attributed to two primary factors. Firstly, the accuracy of prenatal ultrasound diagnostics varies considerably, owing to challenges in obtaining standardized fetal cardiac ultrasound images and interpreting results effectively (8–10). Secondly, the multidimensional nature of prenatal data poses challenges in achieving a comprehensive diagnosis (11, 12).

Obtaining standard views for the prenatal diagnosis of CHD is a complex and uncertain task. This is due to the small size, the fast beating of the fetal heart, the fetus rotating in utero, and the maturity of its structure with gestational age (13). To achieve accurate diagnostic views, the operator must thoroughly understand cardiac anatomy. Additionally, they need a strong spatial imagination and must adjust spatial relationships based on fetal position and gestational age. Therefore, this task poses a challenge for most prenatal screening physicians.

Moreover, diagnosing CHD involves processing highly complex multidimensional information, including organ structure, gene ontology, maternal disease, and clinical pathways. These pieces of information are related to each other, affect each other, and jointly impact prognosis. The weight of each information on prognosis is also different. Furthermore, there are more than 200 sub-types of CHD with significant differences in outcomes and more than 400 known causative genes for CHD (14). At the same time, many potential causative genes and many kinds of maternal diseases are related to fetal cardiac defects, such as autoimmune diseases, gestational diabetes, and infectious diseases. Changes in any variable can lead to changes in clinical decision-making. For example, if a fetus presents with a straightforward ventricular septal defect (VSD), and without additional anomalies, the prognosis tends to be favorable. Contrastingly, when a VSD coincides with chromosomal abnormalities, like the deletion of chromosome 5’s short arm leading to “cat’s cry syndrome,” the prognosis becomes grim. In fact, only a minority of these children survive into adulthood, often exhibiting evident intellectual disabilities (15).

The application of AI in aiding medical diagnosis is growing as it offers fast processing, improved accessibility, and enhanced work efficiency. This has led to a greater demand for AI in clinical settings. Recent advancements in AI techniques have led to numerous studies on CHD, aimed at optimizing the scanning process, improving image quality, and enhancing screening and diagnostic capabilities (16–18). There are many data sources and clinical decision-making processes in these applications. Moreover, numerous AI-based methods have been proposed for analysis specific to different data types. Although several review articles have been written on the application of AI in CHD, these articles focused on either the whole field of cardiology (19–22) or on other single-imaging modalities such as ultrasound images (23, 24). Also some specific tasks like image segmentation have been summarized (25), there is a lack of overall review analysis of AI for CHD. This article addresses the absence of a comprehensive review analysis of AI for CHD by reviewing existing AI-powered applications. It aims to assist beginners and non-specialists in gaining a better understanding of this relatively new technique while also promoting further investigations and applications in the field.

In this review, we introduce the data sources and clinical decision-making processes in CHD that can be addressed with AI methods and discuss the different applications of AI for CHD. We start by presenting AI concepts and illustrating the prenatal diagnosis of CHD in imaging that AI algorithms can currently perform. Then, we present how CHD genetic prenatal diagnosis knowledge can be introduced into AI applications through analytical modeling. Next, we demonstrate the AI applications of underlying factors associated with the development of CHD. Finally, we discuss the remaining challenges to the widespread use of AI in CHD.

## 2 AI concepts

For over two decades, AI has been integral to advancements in the medical field, playing a significant role in developing computer-aided diagnosis, treatment, and decision-making that assist clinical physicians (26). Machine learning is a branch of artificial intelligence that gradually improves statistical methods with increased data to obtain the best model and predict unknown conditions. The basis of machine learning lies in big data, and its abundant resources come from a vast database accumulated in routine clinical practice. Machine learning algorithms require training data to acquire knowledge of the parameters involved in a particular task. Additionally, validation data is often used to optimize these parameters. The accuracy of the task is then assessed using test data that was not previously seen during the learning process. Three types of machine learning methods exist: supervised, unsupervised, and semi/weakly-supervised learning (27). Labeled training data establish the relationship between input and output in supervised learning. For example, the input often consists of images or videos, clinical information (such as maternal factors including age, occupation, medication exposure and mental stress), and gene data. Human annotations can determine the ground truth for supervision, CHD fetal autopsy or postnatal validation, and clinical CHD phenotype. In unsupervised learning, the algorithm clusters unlabeled data to find inherent similarities, and only input data is used without additional annotations. Therefore, a larger amount of data is typically required. Semi/weakly learning aims to achieve the same results as supervised learning while using the minimum amount of labeled data possible. Human annotation is usually a challenging and time-consuming task that can also be influenced by observer dependence. Hence, minimizing the need for annotation can reduce annotation costs and improve the model’s potential for generalization.

Since 2015, AI-powered prenatal diagnosis of CHD has witnessed substantial growth, with a focus on improving data acquisition and assisting tools to enhance screening and diagnostic capabilities. Unlike static organs and adult hearts, prenatal diagnosis of CHD presents specific challenges such as data collection through the maternal uterus, rapid pulsation, small size, and organ maturation during gestational age. The application of AI technologies provides clinicians with convenient tools. For instance, semantic segmentation aids in identifying cardiac structural anomalies, facilitating easier diagnosis for medical professionals. Image classification helps doctors quickly locate the desired fetal cardiac views, while key-point detection assists in obtaining more accurate structural measurement data.

**Figure 1:**
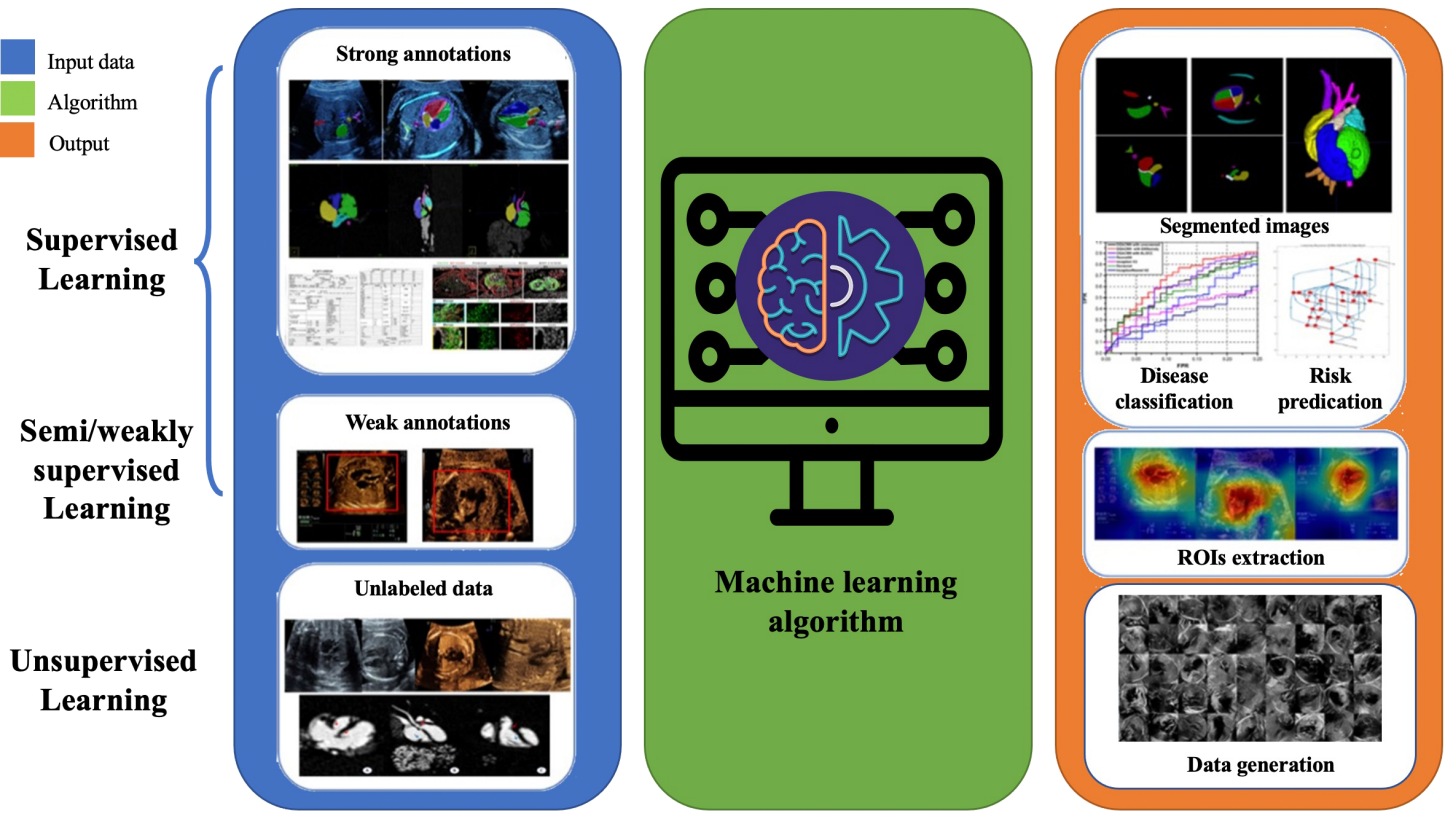
Pipeline of supervised, semi/weakly-supervised and unsupervised learning applications in congenital heart disease.

The primary tasks involved in AI-based prenatal CHD diagnosis encompass CHD imaging diagnosis, CHD-related genetic diagnosis, and analysis of prenatal risk factors associated with CHD (Figure 1). In the following, we will review the major progress made in these areas.

## 3 AI-based prenatal diagnosis of CHD in imaging

**Figure 2:**
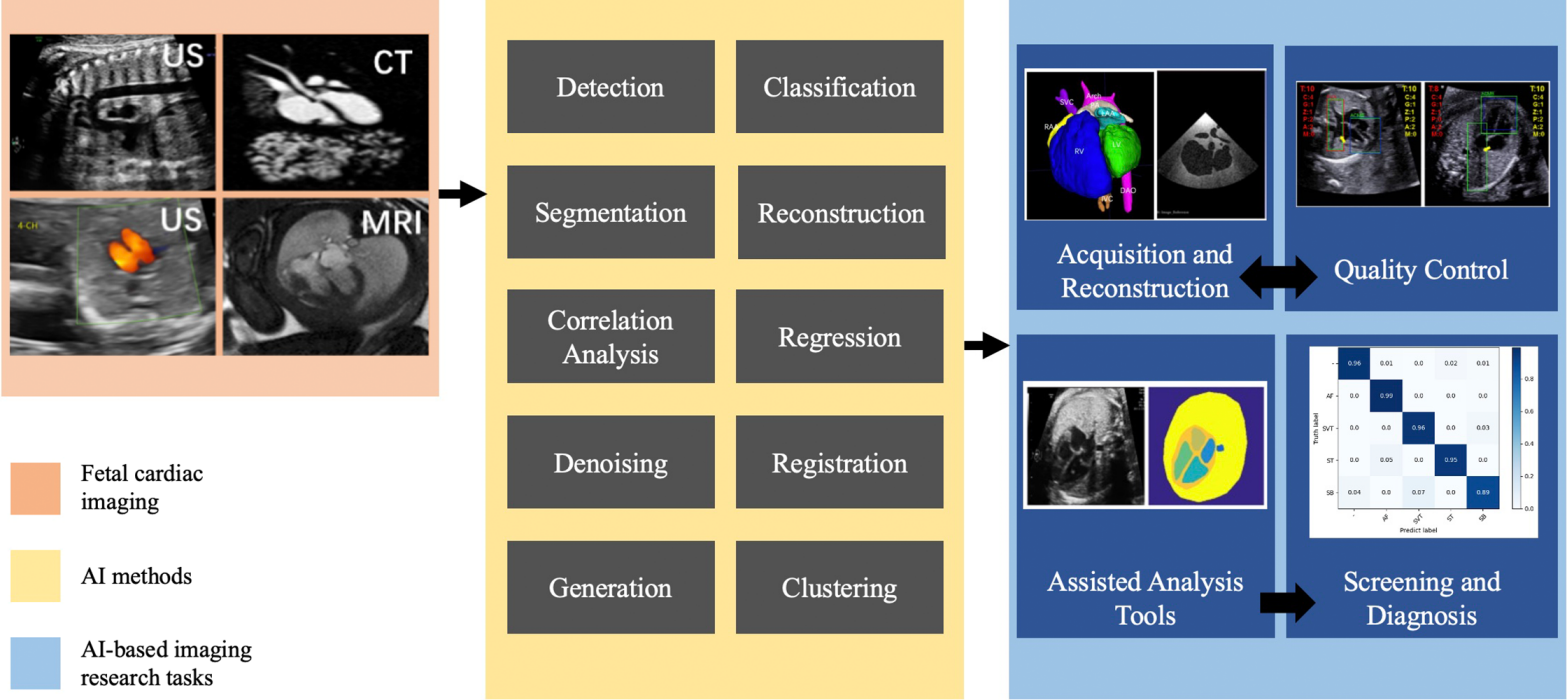
AI-supported algorithms and clinical tasks of prenatal diagnosis of CHD in imaging.

AI methods necessitate four research tasks for prenatal diagnosis of CHD in imaging: acquisition and reconstruction, quality control, assisted analysis tools, and screening and diagnosis. Figure 2 illustrates the interrelationship among these tasks.

### 3.1 Acquisition and reconstruction

AI in fetal heart image acquisition and reconstruction tasks aims to improve the quality of collected images and decrease the time required to obtain different medical images. For example, Yoo et al. (28) introduced unsupervised networks to reconstruct the continuous variation in dynamic MRI sequences with high spatial resolution. Roy et al. (29) combined compressed sensing and a metric-optimized gating network to accelerate imaging of the fetal heart. Uus et al. (30) proposed a deformable reconstruction method for nonrigid motion correction on fetal MRI that can be used for high-resolution reconstruction of the fetal heart. Furthermore, van Amerom et al. (31) proposed a fetal MRI acquisition and reconstruction strategy to improve the quality of reconstructed images and increase the visibility of the small and dynamic anatomical structure of the fetal heart. Due to limitations on radiation exposure during pregnancy, invasive examinations such as CT scans cannot be performed. To achieve three-dimensional imaging of the fetal heart, Yang et al. (32) proposed a workflow that combines post-mortem fetal heart and cardiovascular casting with CT scanning and fetal echocardiography. The process includes obtaining standard views for alignment and then performing a three-dimensional reconstruction.

### 3.2 Quality control

Quality control of fetal cardiac images is crucial for an accurate diagnosis of CHD in the clinic. However, manual quality assessment relies on operator experience and is prone to various limitations, such as labor intensiveness, inconsistencies between observers, and non-standard plane acquisition. Hence, automatic quality assessment plays a vital role in CHD diagnosis. Currently, quality control for fetal echocardiography mostly focuses on selecting standard planes, including defining the integrity of structures and image clarity. For example, Chen et al. (33) initiated the AI analysis of prenatal ultrasound images that employed domain-transferred deep convolutional neural networks to identify the fetal abdominal plane in prenatal ultrasound images precisely. Dong et al. (34) proposed an automatic quality control framework to achieve a standard four-chamber view for fetal cardiac ultrasound, improving the efficiency of CHD diagnosis. Baumgartner et al. (35) also proposed a method to detect standard views of fetuses using 2D US data and critical key anatomical structures on the plane. Chen et al. (36) explored a composite neural network to detect standard planes from fetal heart US scan videos; they also introduced a multitask learning framework to share knowledge across three detection tasks to address the issue of insufficient training datasets.

### 3.3 Assisted analysis tools for CHD

Recently, many studies have focused on tools to assist with CHD, such as segmentation of the fetal heart structure, extraction of the fetal heart rate, and valve motion recognition. Xu et al. (37) a cascaded convolutional neural network was proposed to segment seven anatomical structures and identify useful clinical indicators for prenatal sonographic examination. Wang et al. (38) presented an automated cardiac time interval measurement method for modified myocardial performance index calculation and achieved good results compared with those of expert sonographers. Fernando et al. (39) presented a solution for measuring heart rate variability using Doppler US images. More specifically, a multiple signal characterization algorithm has been applied to estimate the frequency and amplitude of the signal and the variance in the noise component of the signal. However, major limitations exist; the training data in these studies were all from healthy fetal hearts and cannot be applied to cardiac anatomical structures due to CHD.

### 3.4 Screening and diagnosis of CHD

The screening and diagnostic tasks for CHD primarily focus on AI analysis of fetal echocardiography, which can be broadly categorized into classification problems and segmentation of key markers. Gong et al. (40) initially incorporated abnormal data into the AI analysis task, suggesting the application of DGACNN for CHD recognition, utilizing unlabeled video slices to share network weights and achieving favorable classification outcomes. Arnaout et al. (12) conducted multicenter research proposing ensemble classification models to screen for CHD using fetal echocardiograms. The research utilized 1,326 retrospective fetal scans to train ensemble networks, distinguishing normal hearts from those with CHD, and achieved high accuracy and sensitivity for both internal datasets and external imaging. Wang et al. (41) achieved automatic diagnosis of fetal total anomalous pulmonary venous connection by measuring key parameters by segmenting various structures in fetal echocardiography.

## 4 AI-based related genetic diagnosis of CHD

Nearly 20% of fetal cardiovascular diseases are associated with chromosomal abnormalities (42–44). Clarifying the genetic causes has a significant impact on genetic counselling and fertility guidance for the affected couples. Nonetheless, research on the genetic pathogenesis of CHD is limited by the number of family samples and the depth of genetic testing. According to the PubMed database, fewer than 20 non-case reports on whole-exome sequencing for CHD were published before December 2022, with a total sample size of fewer than 1,000 cases. Only 9 studies had a sample size greater than 30; the maximum sample size was only 235 cases (45, 46). Only a few studies using AI-based tasks with CHD genetic test data have been found. Radhakrishna et al. (47) employed logistic regression analysis to select candidate markers for VSD prediction and explored the mechanism of CHD. Sun et al. (48) evaluated the association of maternal genes with the risk of CHD with 569 eligible cases, but due to the limited sample size, the specific subtypes could not be examined. Li et al. (49) suggested the possibility of discerning the nonlinear relationship between genes and diseases from graph convolutional neural networks by considering both the network topology and multiple sources of information on diseases and genes.

## 5 AI-based risk prediction for CHD

A strong association exists between the incidence of CHD and exposure to risk factors, such as maternal illness, medication intake, and mental stress during early pregnancy (50–52). Nevertheless, the underlying mechanism of CHD appears to be multifactorial. Therefore, identifying potential risk factors for CHD through AI is crucial to reduce its incidence. Yang et al. (53) investigated the association between maternal exposure to air pollution and CHD. They used a logistic regression model and demonstrated that maternal exposure to any air pollutants during the first trimester is associated with increased odds of CHD. Wern et al. (54) calculated odds ratios (ORs) by comparing the live birth rates of babies born to mothers with and without diabetes. It also shows the risk of CHD for the fetuses of pregnant women with diabetes was five times greater than the fetuses of pregnant women without diabetes. According to Wurst et al. (55), the use of paroxetine and other psychotropic drugs during pregnancy increases the risk of CHD in population studies and clinical randomized trials. Karatza et al. (56) collected data on the maternal and infant characteristics of fetuses diagnosed with CHD and found that the risk of CHD increased by 2.7 times when pregnant women were exposed to smoking during pregnancy and that the risk increased with increasing smoking dose. However, more than 300 parameters can be considered fetal factors, and the teratogenic factor prioritization problem in CHD remains elusive due to the large search space and insufficient training datasets.

## 6 Remaining AI challenges in prenatal diagnosis of CHD

Prenatal Diagnosis of CHD with AI faces challenges similar to other medical AI applications. Its opaque decision-making process raises interpretability concerns crucial for clinical decision-making. Addressing ethical and legal issues, such as privacy and discrimination, requires clear guidelines. Implementing AI systems demands significant resources and may exacerbate healthcare disparities. Continuous adaptation is essential to align AI models with evolving medical knowledge. Collaborative efforts among healthcare providers, researchers, policymakers, and ethicists are crucial for responsible and ethical AI use in enhancing prenatal care and CHD diagnosis.

Additionally, current AI studies in CHD primarily replicate existing methodologies, such as classification, segmentation, and detection, relying heavily on expert annotations. They lack exploration and optimization of decision-making pathways. These studies can be summarized into three main issues: volatility, insufficiency, and independence (VII).

### 6.1 Volatility

Subjective cognition of prenatal diagnosis of CHD varies greatly, resulting in high volatility among AI models (12, 35). Therefore, forming large-scale and high-quality sample data is the cornerstone of AI training on CHD. Converting task-driven data into data-driven data, particularly by extracting insightful metadata, including deep features, using AI methodologies, enhances the clarity and richness of data acquisition. This increases the likelihood of uncovering deeper associations among the same disease. Additionally, adopting inductive learning approaches from repetitive data paves the way for promising research avenues.

### 6.2 Insufficiency

The data used for AI training in CHD are inadequate for several reasons. Firstly, there is data loss, including genetic and maternal data loss. Additionally, there is a lack of research data standards for CHD. Moreover, due to the unique nature of pregnancy, radioactive scans cannot be conducted without a definitive gold standard for three-dimensional relationship data of the fetal heart. Furthermore, studies on CHD are predominantly one-dimensional, which limits the ability to achieve a comprehensive disease diagnosis and accurate evaluation of maternal and fetal prognoses. Consequently, pursuing multidimensional data fusion, such as the fusion of multi-sectional images of fetal heart, and multi-modal data fusion including ultrasound data and structured data. Additionally, developing inference models based on partial default data shows promise as a fruitful avenue for research.

### 6.3 Independence

More research is needed on the continuity of decision-making. Current solutions focus on single-point solutions, where each piece is an independent problem. Moving forward, this requires viewing it as a cohesive whole, where each piece is interconnected, and the resolution of one piece affects subsequent solutions. Treating the entire process as a system is essential to optimize the decision-making process further. For instance, in acquiring fetal standard ultrasonic sections, factors such as the fetal condition, current probe position, existing sections, and areas of interest need to be considered. AI can optimize the path, guiding operators in the next movement of the probe, ultimately achieving the optimal path for acquiring standard ultrasound sections. Therefore, addressing the optimization of the clinical decision-making process for CHD is a promising research direction.

## 7 Conclusion

CHD has a high incidence and includes a wide spectrum of diseases, as well as a wide range of outcomes. This involves multi-source and multidimensional diagnostic information, introducing both challenges and opportunities to AI analysis of CHD. Current AI research for prenatal CHD diagnosis implies that it can improve the workflow of CHD screening and diagnosis. It can also increase the confidence of prenatal ultrasound diagnosticians and improve the effectiveness of prenatal CHD screening. However, the previous AI paradigm cannot meet the current clinical needs of CHD due to VII problems.

Addressing the challenges in prenatal CHD diagnosis, AI techniques can be utilized to extract deeper-level data features and integrate them into data annotations to improve data re-usability. Additional dimensions, such as sensor location and physician attention data, can be incorporated to enrich the dataset. Furthermore, optimizing clinical decision-making with AI algorithms can enhance efficiency and accuracy. As a result, AI and CHD form a bidirectional interaction cycle, requiring efforts on both the clinical and AI sides. AI drives clinical development in CHD, and CHD drives AI.
